# Germination responses of native fire ephemerals of Patagonian grasslands to smoke water and karrikinolide

**DOI:** 10.3389/fpls.2025.1550692

**Published:** 2025-02-19

**Authors:** Sofía Gonzalez, Jorgelina Franzese, Luciana Ghermandi

**Affiliations:** Institute of Research in Biodiversity and Environment, National Scientific and Technical Research Council, National University of Comahue, Bariloche, Argentina

**Keywords:** fire-related cues, KAR 1, native herbs, seed germination, semi-arid steppe, smoke water

## Abstract

Plant fire ephemerals are well-adapted to fire-prone environments, with germination strategies linked to fire-related cues like smoke. Germination requirements linked to fire cues in Patagonian fire ephemerals are poorly studied, with no research on the effects of smoke water (SW) and smoke isolated karrikinolide (KAR) on their germination. We assessed the germination responses of two native ephemeral herbs, *Boopis gracilis*, and *Nicotiana linearis*, to SW and KAR_1_ at three concentrations (1/100, 1/1000, and 1/10000) and a Control (no SW or KAR_1_). Seeds collected on different harvest dates were incubated in a germination chamber, and germination responses were analyzed using Generalized Linear Models and Kaplan-Meier survival curves. KAR_1_ significantly enhanced and accelerated germination in both species, with the strongest effect at the highest concentration (1/100). In contrast, SW did not stimulate germination and inhibited germination in *N. linearis* at the highest concentration. This inhibitory effect decreased with storage time, suggesting that toxic compounds in SW degrade over time. Additionally, *N. linearis* seeds exhibited variable germination across harvest years, likely due to after-ripening requirements. Our findings highlight the contrasting effects of SW and KAR_1_ on seed germination; KAR_1_ being a reliable germination stimulant. The chemical complexity of smoke water (heterogeneous composition and uncertain concentration) and the time elapsed since its production likely limit its efficacy in promoting germination. These results provide valuable insights into post-fire seedling dynamics in Patagonian grasslands for the conservation in fire-affected ecosystems.

## Introduction

Non-sprouting plant species mainly persist in post-fire communities through seedling recruitment from soil seed banks (Keeley and Fotheringham, 2000; Keeley et al., 2011a). Fire provides a greater opportunity for seedling establishment by releasing resources (e.g. space, light, and nutrients), eliminating pathogens and allelopathic compounds, and reducing competition (Van Staden et al., 2000; Dixon et al., 2009). Additionally, fire can break seed dormancy through fire-related cues, primarily heat shock and smoke, thereby stimulating germination (Keeley and Fotheringham, 2000). The positive response of species to fire cues may confer a significant advantage to seedlings, allowing them to benefit from the post-fire flush of resources, thereby ensuring their establishment (Crosti et al., 2006; Rice and Ross, 2013).

Plant-derived smoke plays an important role in enhancing seed germination of species in fire-prone communities in Mediterranean-type climates (Keeley et al., 2011b). For example, smoke-stimulated seed germination has been observed in five Asteraceae species in Australia (Merritt et al., 2006) and 25 Californian chaparral species (Keeley and Fotheringham, 1998). In South Africa, a significantly higher number of seedlings of *Audounia capitata* (Bruniaceae) were found in smoke-treated areas (de Lange and Boucher, 1990). Smoke water also played a clear role in seedling recruitment of annual species, increasing seedling emergence and establishment in treated soils within the Mediterranean Basin (Tormo et al., 2014).

Smoke can be applied to seeds as an aerosol or as a liquid generated by bubbling smoke through water (Flematti et al., 2004). Germination response to smoke varies greatly among species (Light et al., 2002; Baker et al., 2005a; Buhk and Hensen, 2006), with some species experiencing inhibited germination (Pennacchio et al., 2007; Gómez-González, 2008). Germination inhibition generally occurs with high concentrations or prolonged exposure to smoke (Pennacchio et al., 2007). Because of this species-specific response, it is necessary to conduct experiments over a wide concentration gradient of smoke (Dixon et al., 1995; Lloyd et al., 2000).

Smoke contains thousands of different compounds, and many attempts have been made to identify the active compound responsible for stimulating germination (Baldwin et al., 1994; Van Staden et al., 1995; Dixon et al., 1995). In 2004, two independent studies isolated an organic molecule from the smoke of burning plant material, known as 3-methyl-2*H*-furo[2,3-*c*] pyran-2-one (1) (karrikinolide or KAR_1_) (Flematti et al., 2004; van Staden et al., 2004). KAR_1_ is highly active, heat-stable, long-lasting, water-soluble, and very effective in triggering seed germination and seedling growth at extremely low concentrations (< 1 nM) (Flematti et al., 2004; van Staden et al., 2004; Stevens et al., 2007; Light et al., 2009). Unlike smoke, KAR_1_ solutions are not inhibitory at high concentrations (Flematti et al., 2004). KAR_1_ functions similarly to gibberellic acid by altering the sensitivity of seeds to light, thereby enhancing seed germination (eg. *Angianthus tomentosus*, *Myriocephalus guerinae*, and *Podolepis canescens*) (Merritt et al., 2006). Several subsequent studies demonstrated the presence of other compounds in smoke that promote germination, including five related karrikins (KAR_2_-KAR_6_) (Flematti et al., 2009) as well as cyanohydrins (Flematti et al., 2011). Consequently, karrikins are considered an important family of plant growth regulators (Chiwocha et al., 2009; Nelson et al., 2012).

In the semi-arid steppe of Northwestern Patagonia, dominated by grass-shrub vegetation, fire plays an important role in the structure and function of the ecosystem (Ghermandi et al., 2004). Paleo-climatic studies indicate that fire has been present in Patagonia for at least 10,000 years (Bianchi, 2000). These grasslands exhibit resilience due to the rapid regrowth of grasses and shrubs, as well as the early post-fire mass seedling recruitment of annual native ephemeral herbs from the soil seed bank (Gonzalez and Ghermandi, 2008; Ghermandi et al., 2013). Fire ephemerals are a functional group of plants found in many fire-prone regions of the world such as California (Keeley et al., 2005), South Africa (Keeley and Bond, 1997) and Australia (Baker et al., 2005a, 2005b). Referred to as “the phantom community” by Ghermandi et al. (2004), these species can recruit in great abundance after fires, grow rapidly, produce seeds, and disappear from above-ground vegetation, with their seeds remaining in the soil bank until another fire occurs. They are often confined to heavily disturbed areas such as roadsides, where the dark soil, with its low albedo, absorbs ultraviolet rays, heating the soil and raising its temperature above that of the air at ground level (Matthias et al., 2000). Moreover, in these habitats, summer surface temperatures are like those found at 1 cm in depth during fires (Franzese and Ghermandi, 2012).

Unlike other fire-prone communities worldwide, the germination requirements associated with fire-cues for Patagonian fire ephemeral species have been scarcely studied (Gonzalez and Ghermandi, 2012). These authors reported that the germination of the herb *Boopis gracilis* (Calyceraceae) was stimulated by aerosol smoke combined with heat-shock, while seeds of *Nicotiana linearis* (Solanaceae) were not stimulated by either smoke or heat-shock. These species contribute to the post-fire increase in native species richness and diversity in grasslands (Ghermandi et al., 2004; Gonzalez et al., 2010). Understanding the germination behavior of fire ephemerals could be helpful in developing conservation and management strategies in post-fire grasslands and may provide insights into how these species can be used for restoration purposes. Our objective was to assess the effects of different smoke water and karrikinolide (KAR_1_) concentrations on the seed germination of two fire ephemeral species in NW Patagonian grasslands. We hypothesized that smoke water and karrikinolide solutions enhance the germination, but that the response is species-specific and dependent on the concentration of the solutions. Our study will contribute with valuable knowledge to understanding post-fire dynamics of Mediterranean environments, where fire ephemerals contribute.

## Materials and methods

### Study area

**Figure 1:**
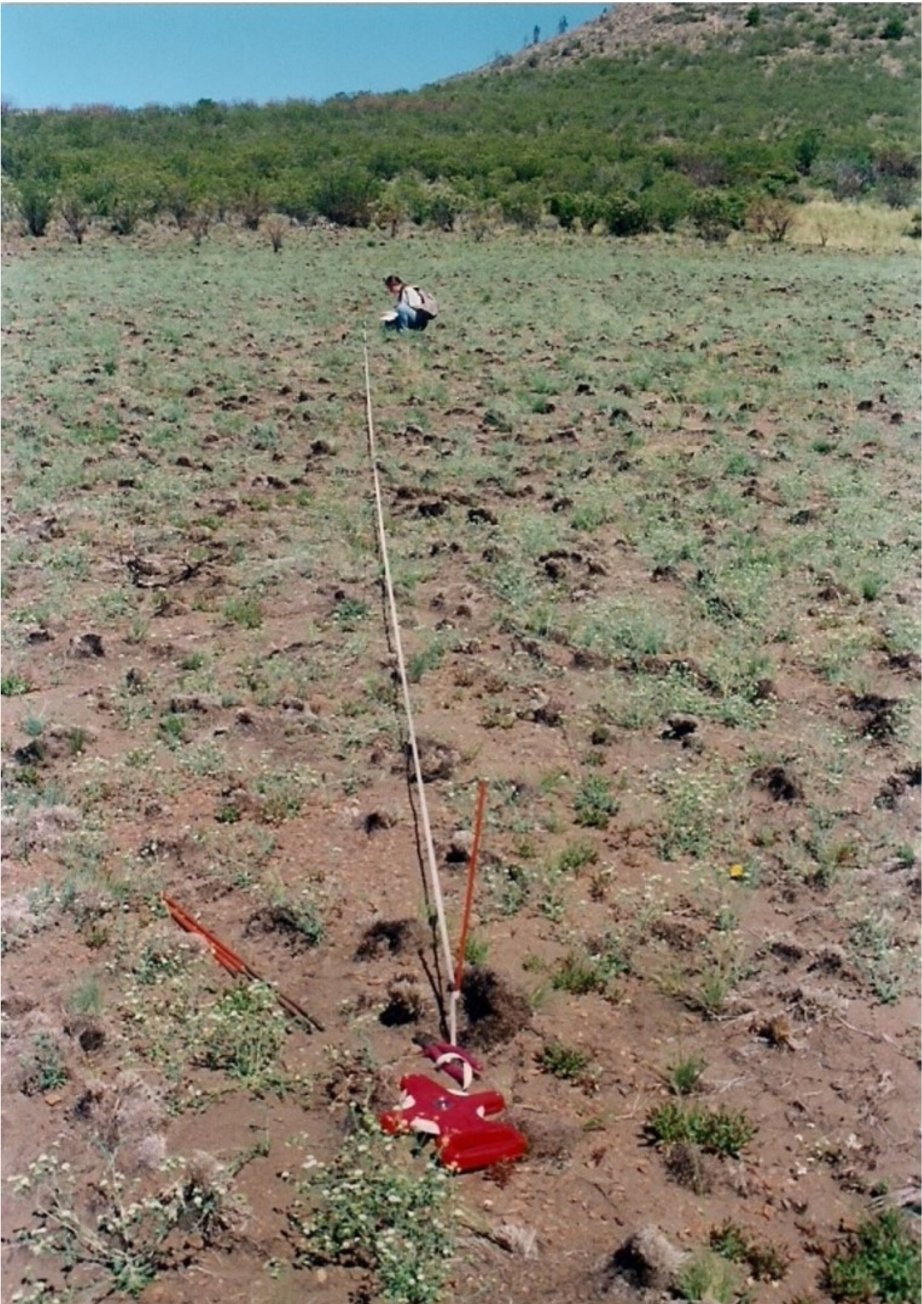
Massive post-fire establishment of the native fire ephemeral *Boopis gracilis* in northwestern Patagonian grasslands.

We collected seeds of the studied species in sectors of northwestern Patagonia semi-arid grasslands, Argentina (San Ramón Ranch; 41°03´19´´S and 18°01´50´´W). These grasslands were affected by a large-scale fire originated by lightning, caused by a severe drought in 1999 (Ghermandi et al., 2004). Immediately after the fire, we observed a great abundance of seven native fire ephemerals: *Boopis gracilis* Phil, *Nicotiana linearis* Phil, *Camissonia dentata* (Cav.) Reiche, *Montiopsis polycarpioides* (Phil.) Peralta, *Heliotropium paronychioides* A.DC., *Chenopodium scabricaule* Speg., and *Descurrainia pimpinellifolia* (Barnéoud) O.E. Schulz (Ghermandi et al., 2004; Gonzalez et al., 2010) (**Figure 1**), which demonstrated their importance in the postfire succession and motivated the study of the effect of fire cues on their germination.

The climate is temperate with a Mediterranean precipitation regime (60% of the precipitation falls in autumn and winter). Mean annual precipitation is 569 mm, and mean annual temperature is 8.6°C (Meteorological station of San Ramón ranch, 1928-2023, unpublished data).

The vegetation is dominated by the perennial tussock grasses *Pappostipa* sp*eciosa* Trin. et Rupr var. major (Speg) and *Festuca pallescens* (St. Yves) Parodi, and the shrubs *Mulinum* sp*inosum* (Cav.) Pers. and *Senecio bracteolatus* Hook et Arnott (Ghermandi et al., 2004). The vegetation cover is approximately 60%, and the gaps (inter-tussock areas) are colonized by native and exotic herbaceous species (Ghermandi and Gonzalez, 2009).

Fires occur during the austral spring-summer period (October-March), when low precipitation and high temperatures promote highly flammable fuel (de Torres Curth et al., 2008). In this region, the relationship between the ENSO phenomenon and fires, is demonstrated by the coupling between El Niño (rainy, biomass accumulation), La Niña (dry, biomass drying). This coupling resulted in the occurrence of major wildfires at the San Ramón ranch in 1964, 1976, 1989, and 1999 (Ghermandi et al., 2019). Additionally, the trend of longer droughts in northwestern Patagonia has been present since 1991 and climate models for the Andean-Patagonian region predict not only an increase in temperature but also a decrease in precipitation (Ghermandi et al., 2019; Pessacg et al., 2020), which translates to extensive and more severe fires.

### Study species and seed collection

We tested the smoke effect on the germination of two fire ephemeral species; *Boopis gracilis* (Calyceraceae) and *Nicotiana linearis* (Solanaceae). Both species are native annual herbs that recruit massively in the post-fire grasslands and disappear two to three years after the fire but persist in the soil seed bank as they have very small seeds (0.10-0.20 mg) (Ghermandi et al., 2004; Gonzalez and Ghermandi, 2012; Ghermandi et al., 2013).

We collected seeds from 10 randomly selected plants of each species in January 2016, 2018, and 2023 for *N. linearis* and in February 2017 and 2018 for *B. gracilis*. The seeds were dry-stored in paper bags, in darkness, at room temperature (20-25°C), and relativity humidity of 30-40% until the assays. To discard empty seeds, we applied the pressure test using tweezers under a stereoscopic microscope (Borza et al., 2007).

### Experimental design

#### Smoke treatments

We used smoke water and karrikinolide solutions to evaluate the effect of different concentrations on the seed germination of ephemeral species.

We followed the protocol of Flematti et al. (2004) to produce smoke water (SW) from the combustion of green (1 kg) and dry plant biomass (2 kg) of the dominant vegetation of the grassland, inside a 200 L metal drum. The smoke was drawn by suction through 5 L of deionized water for 40 minutes. The resultant dark brown solution (plant-derived smoke water) was stored at 5°C. We produced the smoke water in November 2016 and April 2022, after which we prepared three smoke water concentrations (v/v): 1:100, 1:1000, and 1:10000.

To prepare the karrikinolide solutions, we dissolved 1.5 mg of KAR_1_ (3 methyl-2H-furo[2,3-cpyran-2-one) in 100 mL of deionized water (15 mg/L) (Dr. Gavin Flematti personal communication). We handled KAR_1_ (hereafter KAR) with gloves, safety glasses, and a lab coat. We heated the solution for a better dissolution. The final solution was protected from light with aluminum foil, and we prepared the solution concentrations (v/v): 1:100, 1:1000, and 1:10000.

#### Germination trials

We applied a strict sterilization protocol for the seeds and the materials used in the germination trials to prevent mold contamination and avoid the use of fungicides. Tap water and material (forceps, Petri dishes, pipettes, Whatman No.1 filter paper) were sterilized in an autoclave. The assay was set up under a laminar flow hood to maintain sterility. To ensure surface sterility, seeds were immersed in 0.5% v/v sodium hypochlorite solution for 5 minutes prior to treatments and rinsed three times with the sterilized water through a strainer (Gonzalez and Ghermandi, 2012).

Twenty seeds of each species were placed in 45-mm Petri dishes on two layers of filter paper moistened with 1.5 ml of distiller water (control), smoke water (SW), or KAR solution in different concentrations. The treatments were SW1/100, SW1/1000, SW1/10000, and its Control (no SW), and KAR 1/100, KAR 1/1000, KAR 1/10000, and its Control (no KAR) (10 replicates per treatment for each species). We performed four germination trials. In January 2017, we applied SW produced in November 2016 to seeds of *N*. *linearis* harvested in January 2016. In February 2018, we applied SW produced in November 2106 to seeds of *B. gracilis* harvested in February 2017. In April 2018, we applied KAR to seeds of *B. gracilis* and *N. linearis* harvested in February and January 2018, respectively. Finally, in April 2023, we applied SW produced in April 2022 to seeds of *N. linearis* harvested in January 2022 (**Table 1**). Petri dishes were sealed with a layer of plastic parafilm to reduce desiccation. Finally, seeds were incubated in a germination chamber under controlled temperature and photoperiod 12 h light (30°C)/12 h dark (5°C). Monitoring lasted one month, and the germinated seeds were counted under a stereoscopic microscope without opening the Petri dish every two or three days. The protrusion of the radicle was the criterion for germination. The filter paper was moistened with sterile tap water when necessary.

**Table 1:** Seed harvesting dates, experiment dates, the type of solution applied (SW, smoke water; KAR, karrikinolide) to *Nicotiana linearis* and *Boopis gracilis*, and the production date of smoke water were included.

Species	Harvesting date	Experiment date	Solution type	SW date production
*Nicotiana linearis*	Jan 2016	Jan 2017	SW	Nov 2016
	Jan 2018	April 2018	KAR	–
	Jan 2022	April 2023	SW	April 2022
*Boopis gracilis*	Feb 2017	Feb 2018	SW	Nov 2016
	Feb 2018	April 2018	KAR	–

#### Data analysis

We analyzed the effects of the main factors (smoke water and KAR) and levels (1/100, 1/1000, 1/10000, and Control) on total seed germination by using a two-way ANOVA for *Boopis gracilis*, and by fitting generalized linear models with a Poisson distribution and log link function for *Nicotiana linearis*.

We used Kaplan–Meier curves to describe the probability of seed germination as a function of time (days) of *B. gracilis* and *N. linearis* under smoke water and KAR treatments. These curves also serve as indicators of seed germination speed and rate. The log rank test was used to compare the probability of germination between concentrations for both species. All analyses were performed using R software (R Core Team, 2019). The “glm” function from the *stats* package was used to fit the GLM for total seed germination of *N. linearis*. Pairwise comparisons were performed using the *multcomp* package. Kaplan-Meier curves were constructed using the “survfit” function of the *survival* package, and the “survdiff” function from *survminer* package was employed to assess differences in germination probabilities among treatments.

## Results

The interaction between the main factors (Smoke water: SW and Karrikinolide: KAR) and levels (1/100, 1/1000, 1/10000, and Control) was significant in both species (**Table 2**). This indicates that the effects of SW and KAR solutions on seed germination depends on the level of concentration (**Table 2**).

**Table 2:** Effects of smoke water and karrikinolide (main factors), their different concentrations 1/100, 1/1000, 1/10000, and Control (levels), and their interactions on seed germination of *Boopis gracilis* and *Nicotiana linearis*.

Species	Source	df	Statistic value
*Boopis gracilis*			
	Main factors	1	25
	Levels	3	13
	Main factors x levels	3	5
*Nicotiana linearis*			
	Main factors	2	184
	Levels	3	18
	Main factors x levels	6	116

A two-way ANOVA was performed for *B. gracilis* (with F-statistics), and a generalized linear model with a Poisson distribution was used for *N. linearis* (with Wald chi-square statistics). All effects are significant (p < 0.001).

**Figure 2:**
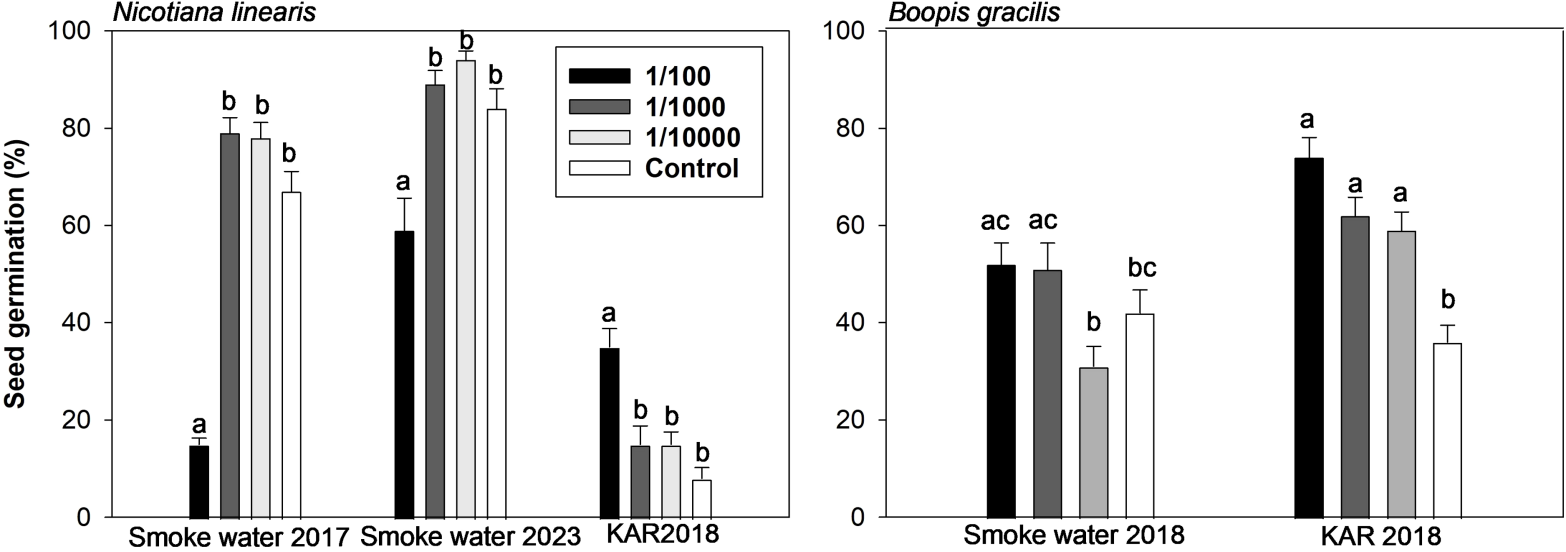
Mean seed germination (%) in response to high (1/100), medium (1/1000), and low (1/10000) concentrations of smoke water and karrikinolide (KAR) solutions for *Nicotiana linearis* and *Boopis gracilis*. For *N. linearis*, experiments were conducted in January 2017 and April 2023 with seeds harvested in 2016 and 2022 and treated with smoke water produced in November 2016 and April 2022. For *B*. *gracilis*, the experiment was conducted in April 2018 using seeds harvested in 2017 and treated with smoke water produced in November 2016. Seeds treated with KAR were harvested in 2018 for both species. Different lowercase letters indicate statistically significant differences.

In *Nicotiana linearis*, germination was negatively affected by the highest SW concentration (1/100) in both years, with a more significant impact on seeds harvested in 2016 (**Figure 2**). Germination was significantly lower in the Control for seeds harvested in 2018 compared to those from 2016 and 2022 (**Figure 2**). In contrast, the highest concentration of KAR (1/100) increased germination 4-fold compared to the Control (**Figure 2**).

In *Boopis gracilis*, germination increased significantly in KAR compared to SW solutions. All KAR concentrations significantly increased germination compared to both the Control and equivalent SW concentrations (**Figure 2**).

**Figure 3:**
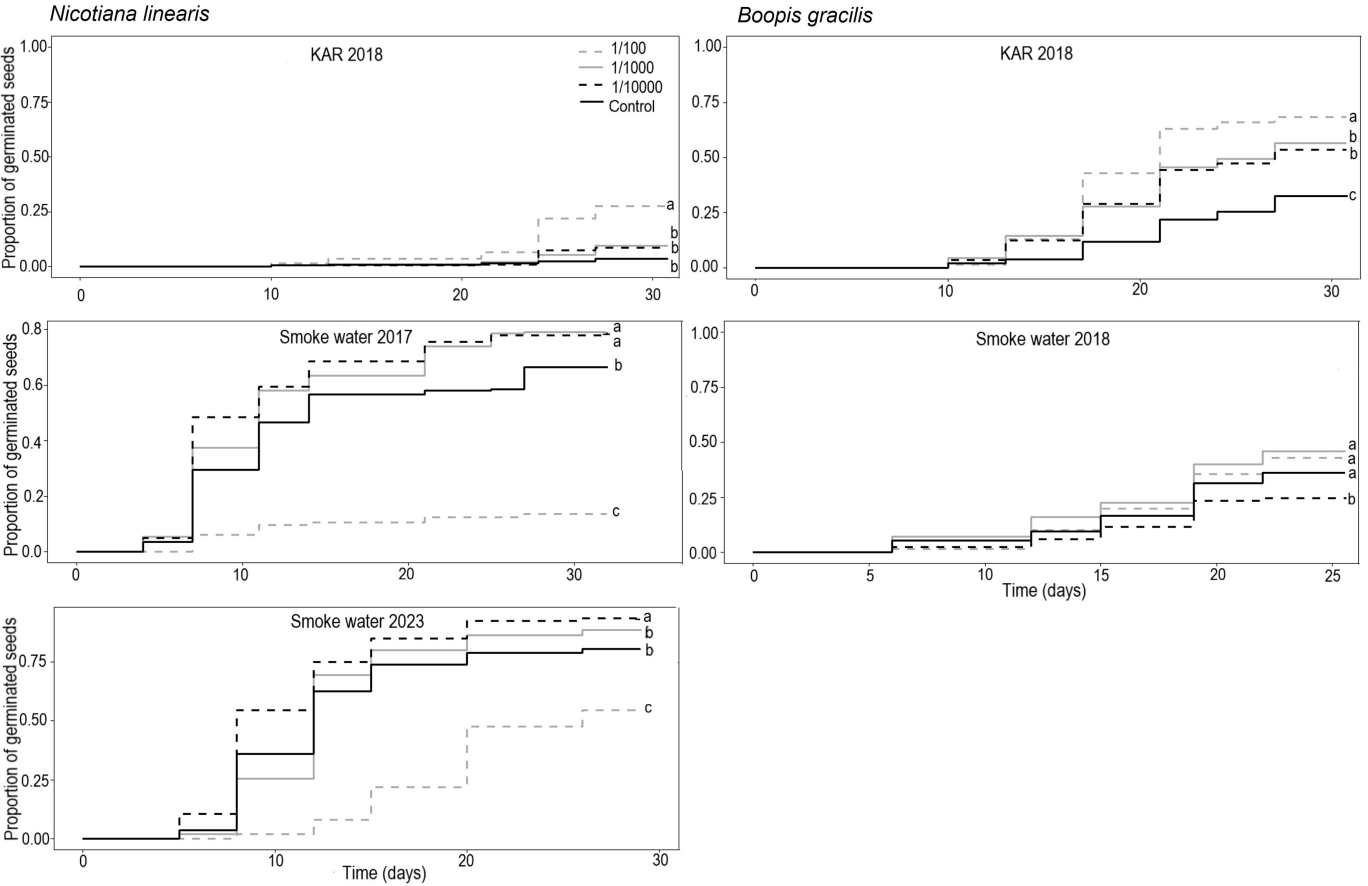
Kaplan–Meier of germination curves for seeds of *Nicotiana linearis* and *Boopis gracilis* at smoke water and karrikinolide (KAR) solutions and concentrations: high (1/100), medium (1/1000), and low (1/10000). Different lowercase letters indicate significant differences among concentrations.

Kaplan–Meier survival analysis of *Nicotiana linearis* showed that the probability of germination was significantly different among KAR concentrations (χ2= 113, df= 7, p < 0.05) and SW concentrations for the 2016 and 2022 harvests (χ2= 113, df= 7, p < 0.05) (**Figure 3**). The highest KAR concentration (1/100) resulted in higher germination probability than the other concentrations and Control (p < 0.001). This concentration also accelerated germination, from day 24, with cumulative germination of 22%, compared to 6% in 1/1000, 8% in 1/10000, and 3% in Control (**Figure 3**; **Supplementary Table S1**). The highest SW concentration (1/100) reduced germination probability in seeds from both 2016 and 2022 harvests (p < 0.001), delaying germination onset and reducing cumulative germination (**Figure 3**).

Seed germination of seeds harvested in 2016 was accelerated at the medium (1/1000) and lowest (1/10000) SW concentrations. Also, the germination probability at both concentrations was higher than that of the Control (p < 0.001) (**Figure 3**). The difference became more pronounced from day 11 onwards, with cumulative germination of 58% in 1/1000 and 60% in 1/10000, compared to 47% in the Control (**Figure 3**; **Supplementary Table S1**). In seeds harvested in 2022, the lowest SW concentration (1/10000) resulted in higher germination than 1/1000 and the Control (p < 0.001) (**Figure 3**), accelerating germination by day 8 day, with cumulative germination of 55%, compared to 26% in 1/1000 and 36% in the Control with similar cumulative germination thereafter (**Figure 3**; **Supplementary Table S1**).

Kaplan–Meier survival analysis of *Boopis gracilis* showed that the probability of germination was significatively different among concentrations in both the KAR (χ2= 113, df= 7, p < 0.05) and SW solutions (χ^2^= 113, df= 7, p < 0.05) (**Figure 3**). In KAR solutions, the probability of germination in 1/100 concentration was significantly higher than those at 1/1000, 1/10000 (p < 0.01), and Control (p < 0.001). The probability of germination at 1/1000 and 10000 was also higher than Control (p < 0.001). All KAR concentrations accelerated germination by day 17,particularly 1/100, which showed 43% cumulative germination compared to 12% in the Control (**Figure 3**; **Supplementary Table S2**). In SW treatment solutions, the germination probability was higher in 1/100, 1/1000, and Control compared to 1/10000 (p < 0.05). This difference started to become noticeable on day 19 when the cumulative germination was 36% in 1/100, 40% in 1/1000, 32% in the Control, and 24% in 1/10000 (**Figure 3**; **Supplementary Table S2**).

## Discussion

Our study shows how the application of smoke water and karrikinolide (KAR) solutions affects the seed germination of *Boopis gracilis* and *Nicotiana linearis*, two abundant post-fire ephemeral native species in semiarid Patagonian grasslands (Ghermandi et al., 2004). KAR enhanced and accelerated the germination in both species, with responses varying by concentration. Both species responded positively to the highest concentration (1/100), while smoke water did not stimulate germination in either species. Additionally, the highest concentration of smoke water (1/100) inhibited germination in *N. linearis*, likely due to the time elapsed between smoke water production and application. *Nicotiana linearis* seeds require after-ripening to release dormancy, as observed in other congeners (Leubner-Metzger, 2005), and KAR application promote and accelerated this process. Our findings are significant for understanding the ecological dynamics of fire ephemerals, which contribute to post-fire increases in species richness and diversity in grasslands (Ghermandi et al., 2004; Gonzalez et al., 2010).

All KAR solutions enhanced seed germination in *B. gracilis*, while only the highest KAR concentration significantly increased germination in *N. linearis*, achieving a fourfold increase compared to untreated seeds. This concentration also accelerated germination, with seeds initiating at 17 days in *B. gracilis* and 24 days in *N. linearis*. Similar processes have been observed in *Nicotiana attenuata*, where germination is regulated by interactions between KARs and phytohormones such as gibberellins (GA) and abscisic acid (ABA). KARs and GA synergistically promote germination, while ABA inhibits it (Schwachtje and Baldwin, 2004; Elsadek and Yousef, 2019); Furthermore, KARs are involved in the early induction of cell-cycle activity, facilitating radicle emergence (Nelson et al., 2012; Naghipour et al., 2016). Rapid germination and establishment facilitated by KARs may confer an ecological advantage in post-fire environments, enabling early colonization and resource capture (Bond and van Wilgen, 1996; Dayamba et al., 2008). Although physiological studies were not assessed in this work, GA and ABA may have influenced the observed germination responses to KAR solutions. Future research should investigate these interactions to clarify their role in promoting rapid germination.

Smoke water failed to stimulate seed germination in either species. Similarly, a previous study found no germination stimulation after exposing these species to aerosol smoke for 60 minutes (Gonzalez and Ghermandi, 2012). However, *B. gracilis* seeds responded positively to a combination of heat shock at 80°C for 5 minutes and smoke (Gonzalez and Ghermandi, 2012). The lack of smoke effect on germination may stem from the complex composition of smoke and the variability in its chemical concentrations (Mojzes et al., 2015). Smoke contains both stimulatory compounds, mixture of chemical compounds such as cyanohydrins, nitrogen oxides, catechol, and the terpenoid 1,8-cineole (Keeley and Fotheringham, 1997; Adriansz et al., 2010; Flematti et al., 2011; Wang et al., 2017; Khatoon et al., 2020), and inhibitory compounds, such as trimethylbutenolide and certain furanones (Light et al., 2010; Gupta et al., 2020).

In evaluating the smoke effect on germination, both commercial liquid smoke (without specification of its components or their proportions) or laboratory-produced smoke water are commonly used, although the methodologies lack standardization. Variations include differences in biomass type and amount, smoke water volume, and the duration of smoke bubbling. For instance, Chumpookam et al. (2012) burned 5 kg of *Oryza sativa* for 45 minutes, to produce 500 ml of smoke water, whereas Gupta et al. (2020) burned 26 kg of grass species to produce 26 l, though combustion time was not reported. Determining the specific compounds in smoke water and their proportions is challenging, and the lack of standardized experimental conditions limits the comparability of results. Our findings can be compared with those of Flematti et al. (2004), as we followed their methodology, although the type of biomass (i.e. different species) we used was different. In contrast, the results obtained with KAR solutions are more directly comparable since they consist of a single substance. We emphasize the need to standardize experimental methods to improve the comparability of findings across studies.

The highest smoke water concentration (1/100) significantly slowed and reduced *N. linearis* germination in both 2017 and 2023. Inhibitory compounds can be toxic at high concentrations or with prolonged exposure (Dixon et al., 1995; Light et al., 2010; Chiwocha et al., 2009; Gupta et al., 2020). Gupta et al. (2020) demonstrated that dilutions of smoke water mitigate the inhibitory effects of these compounds. Our findings support the conclusion that the highest concentration (1/100) exhibits greater inhibition compared to more diluted treatments and that the concentrations of stimulatory compounds are likely low.

The inhibitory effects of the highest concentration (1/100) decreased over time (in 2017, germination of *N. linearis* was 44% lower than in 2023), suggesting that inhibitory compounds may degrade during storage. The smoke water applied in 2017 was produced four months prior to the experiment (November 2016), while the smoke water used in 2023 was produced a year earlier (April 2022). A similar result was observed in *B. gracilis* when smoke water applied in February 2018, produced in November 2016 (with a storage period of one year and three months), did not exhibit inhibitory effects on germination. However, more studies are needed to confirm the degradation of inhibitory compounds in smoke water over time.

The germination percentage of *N. linearis* seeds varied significantly depending on the harvest year. Seeds harvested in 2016 (germinated in 2017), and 2022 (germinated in 2023) showed higher germination compared to seeds harvested and germinated in 2018, suggesting that after-ripening is required for optimal germination. After-ripening involves physiological changes in dry, mature seeds stored at room temperature, promoting dormancy release and enhancing germination (Fenner and Thompson, 2005). In *Nicotiana* species, germination occurs in two steps: testa rupture followed by endosperm rupture, regulated by hormonal dynamics, including the antagonistic interaction between GA and ABA (Leubner-Metzger, 2005; Finch-Savage and Leubner-Metzger, 2006). After-ripening reduces ABA levels, increasing sensitivity to GA and facilitating the sequential rupture of the testa and endosperm (Grappin et al., 2000). Gonzalez et al. (2022) reported higher germination in *N. linearis* of the same seed lot, after two months of incubation compared to this study, while Leubner-Metzger (2005) linked rapid testa rupture in *N. tabacum* to β-1,3-glucanase activation after 60 days of dry storage. Our findings suggest that seeds harvested in 2018 required longer after-ripening to overcome dormancy, leading to lower germination due to insufficient storage time. However, it is noteworthy that the highest KAR concentration (1/100) accelerated this process and increased seed germination.

Our findings enhance the understanding of fire-related cues in post-fire environments, where rapid germination is advantageous. We highlight the contrasting effects of smoke water and KAR on the seed germination of the two fire ephemerals. The chemical complexity of smoke water, its concentrations, and storage time all influence its efficacy in promoting germination, underlining the need for standardized methodologies. In contrast, KAR is a reliable germination stimulant, accelerating dormancy release of *N. linearis* seeds, even with shorter after-ripening periods. Understanding the responses of fire ephemerals to fire-related cues is relevant in fire-affected grasslands, where these species play a significant role in diversity and ecosystem recovery.

## Data Availability

The original contributions presented in the study are included in the article/**Supplementary Material**. Further inquiries can be directed to the corresponding author.
