# Supplementary material for: Germination responses of native fire ephemerals of Patagonian grasslands to smoke water and karrikinolide
Source: Front Plant Sci. 2025 Feb 19;16:1550692. doi: 10.3389/fpls.2025.1550692 (PMC11880283; doi:10.3389/fpls.2025.1550692)
Supplement: Supplementary file 1 [file Presentation1.zip › Table S1.docx]

**Table S1.** Survival curve parameters for Boopis gracilis germination under different smoke treatments (KAR and SW) and concentrations (1/100, 1/1000, 1/10000, and Control). The table includes observation time (days), number of seeds at risk (n°risk), number of events (n°event), cumulative survival probability (survival), standard error (SE), and 95% confidence intervals (lower and upper bounds).

Treatment Time n°risk n°event Survival SE lower 95% CI upper 95% CI

KAR 1/100 10 200 3 0.985 0.0086 0.968 1.000

13 197 23 0.870 0.0238 0.825 0.918

17 174 60 0.570 0.0350 0.505 0.643

21 114 40 0.370 0.0341 0.309 0.443

24 74 6 0.340 0.0335 0.280 0.412

27 68 5 0.315 0.0328 0.257 0.386

31 63 11 0.260 0.0310 0.206 0.328

KAR 1/1000 10 200 9 0.955 0.0147 0.927 0.984

13 191 20 0.855 0.0249 0.808 0.905

17 171 27 0.720 0.0317 0.660 0.785

21 144 35 0.545 0.0352 0.480 0.619

24 109 8 0.505 0.0354 0.440 0.579

27 101 14 0.435 0.0351 0.371 0.509

31 87 10 0.385 0.0344 0.323 0.459

KAR 1/10000 10 200 7 0.965 0.0130 0.940 0.991

13 193 18 0.875 0.0234 0.830 0.922

17 175 33 0.710 0.0321 0.650 0.776

21 142 31 0.555 0.0351 0.490 0.628

24 111 6 0.525 0.0353 0.460 0.599

27 105 12 0.465 0.0353 0.401 0.540

31 93 10 0.415 0.0348 0.352 0.489

Control 10 200 4 0.980 0.0099 0.961 1.000

13 196 4 0.960 0.0139 0.933 0.988

17 192 16 0.880 0.0230 0.836 0.926

21 176 20 0.780 0.0293 0.725 0.840

24 156 7 0.745 0.0308 0.687 0.808

27 149 14 0.675 0.0331 0.613 0.743

31 135 7 0.640 0.0339 0.577 0.710

SW 1/100 6 200 3 0.985 0.0086 0.968 1.000

12 197 17 0.900 0.0212 0.859 0.943

15 180 20 0.800 0.0283 0.746 0.857

19 160 31 0.645 0.0338 0.582 0.715

22 129 15 0.570 0.0350 0.505 0.643

26 114 8 0.530 0.0353 0.465 0.604

SW 1/1000 6 200 14 0.930 0.0180 0.895 0.966

12 186 18 0.840 0.0259 0.791 0.892

15 168 13 0.775 0.0295 0.719 0.835

19 155 35 0.600 0.0346 0.536 0.672

22 120 12 0.540 0.0352 0.475 0.614

26 108 7 0.505 0.0354 0.440 0.579

SW 1/10000 6 200 5 0.975 0.0110 0.954 0.997

12 195 7 0.940 0.0168 0.908 0.973

15 188 11 0.885 0.0226 0.842 0.930

19 177 24 0.765 0.0300 0.708 0.826

22 153 2 0.755 0.0304 0.698 0.817

26 151 12 0.695 0.0326 0.634 0.762

Control 6 200 11 0.945 0.0161 0.914 0.977

12 189 8 0.905 0.0207 0.865 0.947

15 181 14 0.835 0.0262 0.785 0.888

19 167 30 0.685 0.0328 0.624 0.752

22 137 9 0.640 0.0339 0.577 0.710

26 128 11 0.585 0.0348 0.521 0.657
