# Supplementary material for: Germination responses of native fire ephemerals of Patagonian grasslands to smoke water and karrikinolide
Source: Front Plant Sci. 2025 Feb 19;16:1550692. doi: 10.3389/fpls.2025.1550692 (PMC11880283; doi:10.3389/fpls.2025.1550692)
Supplement: Supplementary file 1 [file Presentation1.zip › Table S2.docx]

**Table S2.** Survival curve parameters for Nicotiana linearis germination under different smoke treatments (KAR and SW) and concentrations (1/100, 1/1000, 1/10000, and Control). The table includes observation time (days), number of seeds at risk (n°risk), number of events (n°event), cumulative survival probability (survival), standard error (SE), and 95% confidence intervals (lower and upper bounds).

Treatment Time n°risk n°event Survival SE lower 95% CI upper 95% CI

KAR 1/100 10 200 3 0.985 0.0086 0.968 1.000

13 197 4 0.965 0.0130 0.940 0.991

21 193 6 0.935 0.0174 0.901 0.970

24 187 31 0.780 0.0293 0.725 0.840

27 156 11 0.725 0.0316 0.666 0.790

31 145 15 0.650 0.0337 0.587 0.720

KAR 1/1000 10 200 1 0.995 0.00499 0.985 1.000

21 199 3 0.980 0.00990 0.961 1.000

24 196 7 0.945 0.01612 0.914 0.977

27 189 8 0.905 0.02073 0.865 0.947

31 181 10 0.855 0.02490 0.808 0.905

KAR 1/10000 10 200 1 0.995 0.00499 0.985 1.000

21 199 1 0.990 0.00704 0.976 1.000

24 198 13 0.925 0.01862 0.889 0.962

27 185 2 0.915 0.01972 0.877 0.954

31 183 12 0.855 0.02490 0.808 0.905

Control 10 200 1 0.995 0.00499 0.985 1.000

13 199 1 0.990 0.00704 0.976 1.000

21 198 1 0.985 0.00860 0.968 1.000

24 197 2 0.975 0.01104 0.954 0.997

27 195 2 0.965 0.01300 0.940 0.991

31 193 9 0.920 0.01918 0.883 0.958

SW2017 1/100 7 200 12 0.940 0.0168 0.908 0.973

11 188 7 0.905 0.0207 0.865 0.947

14 181 2 0.895 0.0217 0.854 0.939

21 179 4 0.875 0.0234 0.830 0.922

27 175 2 0.865 0.0242 0.819 0.914

32 173 1 0.860 0.0245 0.813 0.909

SW 2017 1/1000 4 200 11 0.945 0.0161 0.914 0.977

7 189 64 0.625 0.0342 0.561 0.696

11 125 41 0.420 0.0349 0.357 0.494

14 84 11 0.365 0.0340 0.304 0.438

21 73 21 0.260 0.0310 0.206 0.328

25 52 9 0.215 0.0290 0.165 0.280

27 43 1 0.210 0.0288 0.161 0.275

SW2017 1/10000 4 200 10 0.950 0.0154 0.920 0.981

7 190 87 0.515 0.0353 0.450 0.589

11 103 22 0.405 0.0347 0.342 0.479

14 81 18 0.315 0.0328 0.257 0.386

21 63 14 0.245 0.0304 0.192 0.312

25 49 5 0.220 0.0293 0.169 0.286

32 44 1 0.215 0.0290 0.165 0.280

Control 2017 4 200 7 0.965 0.0130 0.940 0.991

7 193 52 0.705 0.0322 0.645 0.771

11 141 34 0.535 0.0353 0.470 0.609

14 107 20 0.435 0.0351 0.371 0.509

21 87 3 0.420 0.0349 0.357 0.494

25 84 1 0.415 0.0348 0.352 0.489

27 83 16 0.335 0.0334 0.276 0.407

32 67 1 0.330 0.0332 0.271 0.402

SW2013 1/100 8 200 4 0.980 0.0099 0.961 1.000

12 196 12 0.920 0.0192 0.883 0.958

15 184 28 0.780 0.0293 0.725 0.840

20 156 51 0.525 0.0353 0.460 0.599

26 105 14 0.455 0.0352 0.391 0.530

29 91 9 0.410 0.0348 0.347 0.484

SW2023 1/1000 5 200 4 0.980 0.0099 0.9608 1.000

8 196 47 0.745 0.0308 0.6870 0.808

12 149 88 0.305 0.0326 0.2474 0.376

15 61 21 0.200 0.0283 0.1516 0.264

20 40 13 0.135 0.0242 0.0951 0.192

26 27 4 0.115 0.0226 0.0783 0.169

29 23 1 0.110 0.0221 0.0742 0.163

SW2023 1/10000 5 200 21 0.895 0.0217 0.8535 0.939

8 179 88 0.455 0.0352 0.3910 0.530

12 91 41 0.250 0.0306 0.1966 0.318

15 50 20 0.150 0.0252 0.1078 0.209

20 30 15 0.075 0.0186 0.0461 0.122

26 15 2 0.065 0.0174 0.0384 0.110

Control 2023 5 200 7 0.965 0.0130 0.940 0.991

8 193 65 0.640 0.0339 0.577 0.710

12 128 53 0.375 0.0342 0.314 0.448

15 75 23 0.260 0.0310 0.206 0.328

20 52 10 0.210 0.0288 0.161 0.275

26 42 3 0.195 0.0280 0.147 0.258

29 39 6 0.165 0.0262 0.121 0.225
